# Dairy Tool Box Talks: A Comprehensive Worker Training in Dairy Farming

**DOI:** 10.3389/fpubh.2016.00136

**Published:** 2016-07-15

**Authors:** Maristela Rovai, Heidi Carroll, Rebecca Foos, Tracey Erickson, Alvaro Garcia

**Affiliations:** ^1^Dairy Science Department, South Dakota State University, Brookings, SD, USA; ^2^Animal Science Department, South Dakota State University, Brookings, SD, USA; ^3^Department of Occupational Safety and Ergonomics, Colorado State University, Fort Collins, CO, USA

**Keywords:** dairy farm trainings, Spanish training, Latino worker, migrant worker, milk quality, dairy sustainability, tool box talks, educational training

## Abstract

Today’s dairies are growing rapidly, with increasing dependence on Latino immigrant workers. This requires new educational strategies for improving milk quality and introduction to state-of-the-art dairy farming practices. It also creates knowledge gaps pertaining to the health of animals and workers, mainly due to the lack of time and language barriers. Owners, managers, and herdsmen assign training duties to more experienced employees, which may not promote “best practices” and may perpetuate bad habits. A comprehensive and periodic training program administered by qualified personnel is currently needed and will enhance the sustainability of the dairy industry. Strategic management and employee satisfaction will be achieved through proper training in the employee’s language, typically Spanish. The training needs to address not only current industry standards but also social and cultural differences. An innovative training course was developed following the same structure used by the engineering and construction industries, giving farm workers basic understanding of animal care and handling, cow comfort, and personal safety. The “Dairy Tool Box Talks” program was conducted over a 10-week period with nine sessions according to farm’s various employee work shifts. Bulk milk bacterial counts and somatic cell counts were used to evaluate milk quality on the three dairy farms participating in the program. “Dairy Tool Box Talks” resulted in a general sense of employee satisfaction, significant learning outcomes, and enthusiasm about the topics covered. We conclude this article by highlighting the importance of educational programs aimed at improving overall cross-cultural training.

## Introduction

Today’s dairy farms are changing dynamically, with increasing herd size and more hired employees. On larger U.S. farms, there is a reliance on non-family immigrant or contract laborers. Latinos have surpassed African-Americans as the nation’s largest minority group, constituting 17% of the U.S. total population in 2014 (1). This is reflected within the dairy industry, and the increasingly Latino workforce requires adapted educational strategies for training. Additionally, the dairy industry sustains high occupational injury rates due to the handling of large animals and highly repetitive tasks demanded from dairy milking parlor workers (2). Lack of effective training strategies creates many knowledge gaps pertaining to both animal and worker health.

A dairy farm involves many day-to-day activities that include animal care, breeding, crop production and feed preparation, cleaning and waste management, and most importantly, milking. Multiple factors interfere with milk quantity and quality (e.g., genetics, environment, and livestock management practices). One of the most costly diseases in dairy farming is intramammary infection or mastitis (3). Bacterial infection is the most common cause of mastitis. High somatic cell counts (SCC) correlate to mastitis and negatively affect milk quality, the cow’s production, and ultimately the profitability of the dairy. Bulk tank milk (BTM) SCC indicate the number of infected animals in the herd as well as the expected decrease in milk yield and quality (4). Mastitis prevalence can be reduced by good cow management, which is established by effective training (5).

In the increasingly immigrant-based large dairy workforce in South Dakota, parlors have an intensive schedule of milking 24 h/day with brief interludes for cleaning. This schedule requires varied shifts, demanding high physical exertion. Many dairy farms, especially those milking cows three times per day, operate by two 12-h working shifts daily. The majority of farm workers have neither basic education nor knowledgeable experience pertaining to dairies (5). In this context, factors, such as socioeconomics, education, cultural diversity, and English proficiency, have enormous impacts on worker understanding of day-to-day tasks, which greatly affect work goals (6).

There is a significant gap in knowledge between dairy professionals and dairy owners regarding employee training. Additionally, employee turnover is costly for most dairy employers. For this reason, the dairy industry needs to restructure the available educational programs for laborers. The engineering and construction industries have successfully implemented targeted innovative employee trainings to improve worker safety and operational efficiency (7, 8). This perspective article focuses primarily on the design procedure of similar innovative training courses by a strategic approach to environmental sustainability, animal health and well-being, milk quality practices, and worker health within the dairy industry. Furthermore, we highlight the potential importance of developing a comprehensive training program in the worker’s native language to address not only current industry standards in dairy farming but also social and cultural differences, leading to a more equitable and sustainable industry for a safe, economical food supply.

## The Training Structure

The study was conducted during a 3-month period, from June until August, 2015, at three commercial dairies in eastern South Dakota, each with 1,600–2,700 Holstein and crossbred Holstein cows (Farm A, Farm B, and Farm C). During the period of study, average milk yield was 33 kg/cow/day, and the monthly bulk milk SCC ranged on average from 159,000 to 270,000 cells/mL.

The target dairy workers were primarily Spanish-speaking Latino migrant workers. Seventy-five people related to milking operations, cow handling, and bedding/hospital pen cleaning participated in the program.

Innovative within the dairy industry, the program was based on trainings used by the engineering and construction industries. Called the “Dairy Tool Box Talks,” weekly trainings were conducted with dairy employees in Spanish. The program talks were conducted over a 10-week period and included a 1-h hands-on with live cattle and eight trainings of 30-min classroom-style covering the following topics:

1. Basic cow and milk production knowledge.
2. Basic cow housing and facilities overview.
3. Animal health and cleanliness: cow signals.
4. Consistent and proper milking procedures.
5. Mastitis and SCC.
6. Safe hands-on cow handling (1 h).
7. Cultural differences within the labor place.
8. Animal welfare and risks of animal organization.
9. Zoonosis and using good ergonomics.

For each session, a PowerPoint presentation was prepared to provide more effective presentations and a better understanding of the topics presented. The use of interactive games, drama activities, and also invited speakers were used to improve the employees’ understanding of the different topics covered during the program sessions. Participants received a one-page handout in Spanish with detailed information on the week’s topic at each session.

A final evaluation session for employee group feedback and certification was conducted at week 10 using Turning Technologies^®^ (data not shown) and flipcharts to interactively convey information and discuss their impressions of the program. Most of the dairy workers involved in the “Dairy Tool Box Talks” participated in the feedback session. At the end of the training period, a feedback session was also provided to the owners, managers, and herdsmen to discuss their impressions of the program and any employee improvements observed.

### Sample Collection, Laboratory Determinations, and Statistical Analysis

Bulk tank analysis is a useful screening tool used to monitor specific problem areas within the dairy farm, identify weak management protocols, and provide useful information on SCC.

For microbiologic activity determination, 50-mL BTM samples were taken on three consecutive days each week and combined. Samples were stored at −20°C until analyzed at the Animal Disease Research and Diagnostic Laboratory at South Dakota State University (Brookings) according to reference microbiology methods for BTM.

Composite weekly samples were cultured on selective media, and colonies were identified using preliminary biochemical and selective media tests. Colonies were grown overnight at 37°C on tryptic soy agar (Remel Inc.) infused with 5% sheep blood or brain–heart infusion agar, mannitol salt agar, MacConkey agar, and modified Edward agar. Samples were identified through matrix-assisted laser desorption/ionization and time-of-flight (MALDI-TOF) mass spectrometry (MALDI Biotyper; Bruker Daltonics Inc., Billerica, MA, USA) when necessary.

Bulk Tank Milk for somatic cell count (BTSCC) were collected daily at each farm by a licensed milk hauler and reported to the researchers. Data of BTSCC and microbiology identification results were log (base 10) transformed to normalize the data before analysis using the PROC MIXED (version 9.3; SAS Institute Inc., Cary, NC, USA). A mixed procedure for repeated measurements was used for BTSCC data and included the fixed effect of farms and time relative to training (weeks 1–10) and the random effect of tank sampling within farm. The mixed model used for the bacteriology identification results included the fixed effects of the farms and the training period, which was classified as start, mid, and final sessions (1–3, 4–6, and 7–10, respectively), and the random effect of tank sampling within farm. Significance was declared at *p* < 0.05, unless otherwise indicated.

## The Learning Innovative Approach

In 2016, the gap between non-fluent English and Native English speaking dairy farm personnel is greater than ever. The inability to communicate has consequences in producing a safe food supply while contributing to the sustainability of the dairy industry. The agriculture sector in the U.S. recognizes that foreign-born workers are increasingly becoming a vital part of the community and the local workforce, especially in the dairy industry. Immigrant workers account for over 50% of all U.S. dairy labor which produces over 79% of the U.S. milk supply (9). It was estimated that 41% of the dairy labor force was immigrants with the majority coming from Mexico (10). Evidence from the American Farm Bureau (AFB) (11) suggests the number of immigrant laborers may be even greater than the estimates.

The owners of large dairy enterprises are business savvy and not the traditional reactive farmers of the past. Today’s proactive owners oversee the health and safety of their employees and ensure consistent, safe care of the cows, which in turn positively impacts the milk production and quality (e.g., minimizing mastitis cases), while also minimizing injuries to workers in this high-risk industry. However, few dairies have active worker training programs that meaningfully educate workers about key principles of livestock care and follow up with evaluations of performance at periodic intervals (12).

The most common source of on-farm training has been the traditional format commonly offered by University Extension Programs. This provides unbiased non-formal education and learning activities to a wide range of people, including agricultural producers and their employees. Other training opportunities through pharmaceutical, nutrition, and reproduction service companies are also offered, but this type of training is biased and limited in duration and impact. The effectiveness and feasibility of training transfer by these two mechanisms is difficult to determine. Non-English speaking employees are hesitant to ask questions during single-session trainings due to their cultural upbringing. Without recognizing this cultural difference within the dairy employees, the one-time training session is an ineffective training tool.

New training approaches implementing appropriate on-farm management practices with short periodic trainings in Spanish will enhance sustainability of the dairy industry by enhancing milk quality, decreasing milk loss, improving dairy cows’ health, and reducing employee injuries/illnesses. Additionally, farms will see improvement in worker’s performance due to a decrease in days off from worker injury or illness and enhanced job performance due to increased knowledge, impacting job performance. Cultural consideration is central to training design and implementation. The “Dairy Tool Box Talks” was designed to provide efficient short-duration hands-on educational demonstrations analogous to tool box talks and pre-task planning in the construction industry. Sessions covered basic modern dairy operation, including basic animal care and handling practices, cow comfort awareness and worker welfare, proper milking protocols, and worker safety. Educational topics focused on preventing zoonosis, managing risks of animal organization, and important cultural differences.

The production of high quality milk is a requirement to sustain a profitable dairy industry, and SCC values are routinely used to identify subclinical mastitis and define quality standards (13). Mastitis is one of the three most significant health problems of the worldwide dairy herds, together with lameness and fertility problems (14) and is the most costly disease of the dairy industry (15). Mastitis has important effects increasing SCC as well as in reducing other milk components (e.g., protein and fat levels and its impact on cheese manufacturing). In the U.S., the legal maximum BTSCC for liquid market (Grade A milk) shipments is 750,000 cells/mL, as outlined in the U.S. Pasteurized Milk Ordinance (16, FDA-PMO, 2011), and a threshold of <200,000 cells/mL is considered to be of the most practical value used to define a mammary quarter as healthy (13). In this research, BTSCC values were recorded each week throughout the training sessions to measure milk for quality indicators and to monitor the dynamics of possible decrease in intramammary infections due to a proper training. Table 1 shows the average BTSCC milk composition for all three farms, where the average values were significantly different (*p* < 0.001) within farms. The difference in BTSCC levels between farms can be explained by individual differences between cows of each farm and by differences in management practices and consistency that may increase the number of intramammary infections within the herd. The observed trends in milk BTSCC for the participant farms were below the accepted threshold for Grade A milk. Farm A and C were within the range of a more healthy herd, whereas Farm B reached values above 250,000 BTSCC on average (Table 1).

**Table 1:** **Average somatic cell (SCC; ×1,000/mL of milk) and bacterial counts (cfu/mL of milk) measured from bulk tank milk (BTM) samples collected throughout the training period**.

Weeks	BTM samples											
	SCC			Total coliforms			Non-agalactiae *Streptococcus*			*Staphylococcus* sp.		
	Farm^a^			Farm			Farm			Farm		
	A	B	C	A	B	C	A	B	C	A	B	C
1	138	224	179	.	100	.	.	1,091	128	.	150	350
2	133	239	169	425	0	525	600	500	24,500	100	30	24,000
3	155	262	199	15	.	0	220	.	2,525	1,050	.	2,000
4	141	252	197	100	.	24,000	322	.	497	347	.	1,850
5	168	271	211	1,050	.	0	1,450	.	8,825	1,400	.	13,500
6	170	281	220	0	.	50	4,300	.	11,500	400	.	86,000
7	147	276	217	5	.	0	2,175	.	2,500	435	.	6,425
8	168	304	206	0	.	215	925	.	1,000	800	.	150
9	173	278	216	15	100	100	105	185	10,350	350	200	0
10	166	279	214	0	200	0	35	1,350	1,500	115	200	450

*^a^Weeks had significant effect (*p* < 0.05) on milk SCC only*.

*“.” indicates missing data*.

The significant increase (*p* < 0.001) in milk BTSCC during the training period can be explained by the weather season. The trainings were done during summer time (warmer and wetter season), which has favorable climatic conditions for microbial growth (17, 18). Together with the animals being more exposed to mastitis pathogens and the evidence that heat stress can negatively affect their immune system, there will be an increase in BTSCC because of their reduced capability to respond to intramammary infections (19).

It seems that there is a correlation between heat stress and decreased immunity in dairy animals. Thompson et al. (20) showed that heat stress in the dry period negatively affected the immune response later in lactation when cows were submitted to a *Staphylococcus* challenge; the heat-stressed cows had lower neutrophils counts and SCC in milk than thermoneutral cows. They also found that heat-stressed cows during the dry period had higher incidence of mastitis later in ensuing lactation. In 2015, the warmest day was June 9 (93°F), and the hottest month was July (average daily high temperature of 81°F), as reported by the National Weather Service records (21). The significant increase in BTSCC is within the range expected for the summer season and probably has no relation with the training sessions.

Identification of pathogens in milk is considered as the definitive diagnosis of intramammary infections in dairy herds, and it is important for disease prevention and control. BTM analysis is a good tool to identify the weak management areas and procedures that are probably being practiced. *Streptococcus agalactiae*, mainly transmitted from cow-to-cow (with contaminated udder wash cloths or teat cups), was not described in any of the farms during the training period (data not shown).

Microbiological identification in the BTM samples is described in Table 1. *Staphylococcus* sp. are often found on cow skin and are transferred to milk *via* poor udder milking preparation. Decreasing counts during training may be associated with increased knowledge of milking preparation, hygiene, and routine consistency. *Staphylococcus* sp. counts varied by farm (*p* < 0.05). Farm C showing the highest counts but, as observed in Table 1, we did not detect a significant effect of the period (*p* = 0.54). Follow-up sampling during subsequent months would have been necessary to determine a significant effect of the training on other milk quality traits.

In Table 1, the results for *non-agalactiae Streptococcus* normally present in teat skin and environment showed no statistical trend for any farm or training period. The bulk tank sampling period only during training may not be appropriate to determine the effectiveness of cow preparation and to evaluate changes made in milking protocols prior to milking. Additionally, the coliform counts did not differ significantly by farm. However, lower counts were observed during the last 4 weeks of training (Farm A and C; *p* < 0.05; Table 1). The number of coliforms in BTM is almost entirely related to skin contamination at the time of milking and to the degree of bedding contamination with coliform bacteria (22). These results may indicate better hygiene practices as indicated in positive feedback from farm A owner and manager.

In the “Dairy Tool Box Talks” lack of motivation to learn or improve working environment were offset by interactive, engaging, and pictographic training. Other incentives were “social time” with employees. (e.g., handouts, pizza, gifts) and a training certificate of completion.

Nearly 70% of the workers were in attendance at the last session and actively contributed showing their appreciation and interest in almost all the topics presented. They showed special interest for mastitis and milk quality, milking procedures, hygiene in general, zoonosis awareness, cultural differences, ergonomics, overall U.S. law and sanctions, and cow handling. The employees also called the program informative and dynamic, expressing desires to continue the learning process with topics not covered, such as farm management, artificial insemination, and calving practices. Nearly 85% of the employees agreed that sessions helped them being more confident in doing their job, and 76% considered the length of the program as adequate. On the other hand, 95% expressed a desire for more owner and managers directly participating during the talks. Remarkably, 95% believed that receiving a training certificate was valuable for their current job or future jobs.

Owners, managers, and herdsmen’s comments included noticeable changes in employee behavior, improved working relations, positive attitude at the workplace, better working performance, and more awareness on hygiene issues. The sessions were highly effective because they were given in the workers’ native language. An overall improvement in milker’s attitudes about the milking procedures was also observed. Other notable observations were that employees moved cows better by being patient, calm, and consistent with them. These changes could increase employee’s productivity, reduce the costly on-the-job accidents caused by uninformed workers, and improve the retention rate. The “Dairy Tool Box Talks” is an excellent example of how employee training programs and manager and/or owner involvement can lead to more effective communication and improved work performance within the dairy.

## Closing Remarks

The feedback of the “Dairy Tool Box Talks” program provided a general sense of employee satisfaction, great learning achievement, and enthusiasm for the sessions. The social challenge in large modern dairy farms is employees’ understanding, awareness, and motivation. Cultural considerations, especially training in Spanish, were key for the success of this program.

The training program was viewed successful by the owners and managers. The topics covered were appropriate and helpful with a format that met the needs of each farm’s schedule and milking shift changes. When owners and managers participated during a session, more positive employee responses were noted about the topic.

In the future, the “Dairy Tool Box Talks” trainings will be offered in the original 10 week period or a shorten version. Further trainings involving other farms should be planned in order to evaluate the potential impact of this pilot program and its contribution to long-term sustainability within the dairy sector. A follow-up survey to evaluate the employees learning achievement along with periodic BTM samples is also needed.

## Author Contributions

Each author made substantial contributions to conception and design of this proposal as well as the acquisition and interpretation of data.

## Conflict of Interest Statement

The authors declare that the research was conducted in the absence of any commercial or financial relationships that could be construed as a potential conflict of interest.
